# Detection of *MET* Polysomy by Next-generation Sequencing and Its Clinical Relevance for *MET* Inhibitors

**DOI:** 10.1158/2767-9764.CRC-22-0438

**Published:** 2023-04-04

**Authors:** Boyang Sun, Tian Qiu, Xiaoling Zeng, Jianchun Duan, Hua Bai, Jiachen Xu, Jin Li, Junling Li, Xuezhi Hao, Yutao Liu, Lin Lin, Hongyu Wang, Xin Zhang, Jia Zhong, Jie Wang, Jianming Ying, Zhijie Wang

**Affiliations:** 1 CAMS Key Laboratory of Translational Research on Lung Cancer, State Key Laboratory of Molecular Oncology, Department of Medical Oncology, National Cancer Center/National Clinical Research Center for Cancer/Cancer Hospital, Chinese Academy of Medical Sciences and Peking Union Medical College, Beijing, P.R. China.; 2 Department of Pathology, National Cancer Center/National Clinical Research Center for Cancer/Cancer Hospital, Chinese Academy of Medical Sciences and Peking Union Medical College, Beijing, P.R. China.; 3 Geneplus-Beijing Institute, Beijing, P.R. China.; 4 Guangdong Provincial Key Laboratory of Translational Medicine in Lung Cancer, Guangdong Provincial People's Hospital/Guangdong Provincial Academy of Medical Sciences, Guangdong, P.R. China.

## Abstract

Next-generation sequencing (NGS) has failed to detect mesenchymal epithelial transition factor gene (*MET*) polysomy in previous studies. We included three non–small cell lung cancer (NSCLC) cohorts in this retrospective study to establish new criteria for detecting *MET* polysomy and to explore the clinical relevance of *MET* polysomy. Cohort 1 included 53 patients whose tissues were available for both FISH and NGS assays. Paired plasma and tissue samples were obtained from 261 patients with NSCLC as cohort 2. Cohort 3 included 46 patients with metastatic NSCLC, who presented with *MET* copy-number gain assessed by NGS. ROC analysis demonstrated that a cut-off point of 2.3 copies achieved the maximum Youden index in discriminating polysomy from normal copy number. Compared with the FISH test for *MET* polysomy, the sensitivity, specificity, and agreement of NGS were 90%, 90%, and 96.2%, respectively. Following optimization using maximum somatic allele frequency, the sensitivity and specificity of NGS for defining polysomy using plasma samples according to different circulating tumor DNA mutation frequencies were 42% and 63%. The concordance rate between tissue and plasma samples for detecting polysomy was 85%. Regarding the response to *MET* inhibitor, the median progression-free survival (PFS) of the *MET* amplification group was significantly higher than that of the polysomy group. The median PFS was similar between the polysomy and normal groups. Our results indicated that NGS may serve as an alternative method for detecting *MET* polysomy in NSCLC tissues. Moreover, patients with *MET* polysomy may not benefit from *MET* inhibitors.

**Significance:**

In this study, we established a methodology to differentiate polysomy from normal copy numbers and amplification using NGS. Moreover, this study suggests that it is critical to discriminate *MET* polysomy from amplification, for the former may dilute the clinical benefit of *MET* inhibitors.

## Introduction

Alterations in the mesenchymal epithelial transition factor gene (*MET*), such as exon 14 skipping mutation and amplification, have been regarded as driver oncogenic mutations and are potentially targetable (1–4). Moreover, *MET* copy-number gain (CNG) has been described as one of the mechanisms of acquired resistance to *EGFR* tyrosine kinase inhibitors (TKI; refs. 2, 5, 6). In the majority of clinical trials, a range of FISH criteria was used to identify patients with *MET* CNG, which could provide information on the absolute *MET* copy number and the *MET*/centromeric enumeration of chromosome 7 (CEP7) ratios (7). However, several limitations exist: this method allows us to investigate only the regions for which FISH probes are available, and multiple FISH probes are needed to be comprehensive, with each probe requiring a resource-consuming validation. Other assays, such as IHC or droplet digital PCR, were neither in poor agreement with FISH nor feasible in the clinic (8–10). In contrast, next-generation sequencing (NGS) technology has improved gene mutation detection and shows many advantages. However, an appropriate threshold for the aforementioned methodology remains to be determined, on the basis of which a *MET*-target therapy would likely be effective (7).

A proportion of cases with *MET* CNG is actually harboring polysomy rather than amplification (11). According to current theories, polysomy is not regarded as a driver oncogenic mutation (12). *MET*/CEP7 ratios were used to discriminate amplification from polysomy and normal copy number using a FISH test. Meanwhile, NGS has failed to separate *MET* amplification from polysomy in previous studies (12, 13), and the detection of polysomy by NGS represents a clinical gray zone. The threshold of *MET* copy numbers to distinguish polysomy from normal copy numbers has not yet been established. Therefore, it remains unclear whether NGS can serve as an alternative method to identify different *MET* abnormalities.

In this study, we aimed to establish an optimal copy-number cut-off value to differentiate polysomy from normal copy numbers using NGS. In addition, we proposed a specific algorithm to detect the *MET* status following NGS, which is capable of discriminating polysomy from *MET* amplification. Moreover, the detection performance of NGS for *MET* polysomy was investigated using both tissue and plasma samples. Furthermore, we explored the response to *MET* inhibitors in patients with different *MET* statuses to determine which type of *MET* status may benefit from *MET* inhibitors.

## Materials and Methods

### Study Participants and Design

Cohort 1 was used to establish the cut-off value of *MET* CNG to distinguish polysomy from normal copy numbers. Between October 2018 and July 2021, 53 patients with NSCLC harboring *MET* CNG were included in this study. Tissue samples of all patients were available for both FISH and NGS assays. An in-house cohort of 155 patients with untreated *EGFR*-mutated NSCLC was used to verify the rationality of the proposed method. In this cohort, 155 patients were diagnosed with metastatic *EGFR*-mutated NSCLC and were untreated previously. All patients harbored a sensitizing *EGFR* mutation, including 67 with *EGFR* 19del, 71 with *EGFR* L858R, 7 with *EGFR* G719A, 5 with *EGFR* S768I, and 5 with *EGFR* L861Q.

Cohort 2 was used to evaluate the concordance of *MET* status between tissue and plasma samples using NGS. Paired plasma and tissue samples were obtained from 261 patients with NSCLC at the same time. The *MET* status was divided into three groups: amplification, polysomy, and normal copy number, according to the cut-off value described above. All 261 patients were allocated to one of three groups based on their *MET* status.

Cohort 3 was used to determine the correlation between the response to *MET* inhibitors and different *MET* statuses. Patients in cohort 3 were diagnosed with advanced NSCLC with a high *MET* copy number (assessed using NGS with tissue samples) and progressed after at least one line of therapy. In total, 46 patients were included in this retrospective study from June 2018 to November 2021.

Supplementary Figure (Supplementary Fig. S1) shows a flowchart of the study design. Written informed consent was obtained from all the patients, and the study was approved by the Ethics Committee of the National Cancer Center/National Clinical Research Center for Cancer/Cancer Hospital, Chinese Academy of Medical Sciences, and Peking Union Medical College.

### FISH

FISH was performed using the Vysis *MET* Spectrum Red FISH Probe Kit and Vysis CEP7 Spectrum Green FISH probe kit. According to the present criteria, *MET* amplification was defined as gene copy number ≥5 and/or *MET*/CEP7 ratio > 2.0.

### Next-generation Sequencing

#### DNA Extraction and Sequencing

DNA was extracted from fresh-frozen tissues or formalin-fixed, paraffin-embedded (FFPE) tumor tissue specimens using the QIAamp DNA Mini Kit (Qiagen) and the ReliaPrep FFPE gDNA Miniprep System (Promega). DNA was extracted from plasma specimens using the QIAamp Circulating Nucleic Acid Kit (Qiagen). All samples were obtained from patients after signing informed consent. Prior to pooling, the samples’ concentrations were quantified by Qbit. A minimum of 15 ng of cell-free DNA was required for NGS library construction. Next, the DNA was sheared into fragments at a 200–250 bp peak using a Covaris S2 ultrasonicator (Covaris, Inc.), and indexed NGS libraries were prepared using the NEBNext Ultra DNA Library Prep Kit for Illumina (NEB). Finally, the 1,021 panel (Integrated DNA Technologies, Inc.), covering approximately 1.5 Mbp of the genome and targeting 1,021 cancer-related genes, was used for hybridization enrichment. The indexed libraries were sequenced using a 100-bp paired-end configuration on a DNBSEQ-T7RS sequencer (MGI Tech) or Gene+Seq-2000 sequencing system (GenePlus-Suzhou), producing 3 Gb of data for fresh specimens/FFPE.

#### Quality Control

Standard quality control was performed on all sequencing data for all samples. A coverage depth of not less than 100X in more than 98% of exons, sequencing depth of at least 200X, and mapping rate of not less than 95% must be achieved.

### *MET* Amplification and Polysomy Analysis

#### Copy-number Variation Analysis

Variant calling and copy-number variation analysis of each capture and non-capture regions were performed using the cnvkit (https://github.com/etal/cnvkit). Genomic DNA extracted from peripheral blood leukocytes in the same batch was used as a background control. On average, we set a bin size of 120 bp for the capture regions and 150 kb for the non-capture regions. Default settings were used for all remaining parameters.

#### Identification of *MET* Amplification and Polysomy

First, the *MET* gene copy number was calculated to determine whether the copy number was increased. *MET* amplification was assessed according to the ratio of CNG to baseline obtained from pooled data from normal samples. Amplified regions on chromosome 7q, including 7q32.1, were searched using the copy number of non-capture regions. A *t* test was used to assess the differences in copy numbers between adjacent chromosomal bands in the non-capture regions on chromosome 7q. *P* values ≥ 0.1 were regarded as not statistically significant. Continuous chromosomal regions were obtained in which there were no significant differences between any two chromosomal bands. Similar adjacent regions were merged when any of the following conditions were met: (i) The proportion of chromosomal bands without significant difference in copy number between smaller and larger regions was higher than 50%. (ii) The larger region contained more than seven chromosomal bands, and the proportion of chromosomal bands without significant difference was higher than 30% in smaller and larger regions. The mean copy number of each region was calculated. CNG was defined as a mean copy number of the region higher than 2.3. The length of the amplified region was estimated as a percentage of the total length of chromosome 7q.

#### Distinguishing *MET* Amplification from Polysomy

If the *MET* copy number was increased and the copy number of the region including 7q32.1 did not increase or the region with increased copy number accounted for <10% of the length of 7q, the *MET* gene was defined as amplified. If the copy number of the region including 7q32.1 was increased and the region with increased copy number accounted for >10% of the length of 7q, it may be defined as *MET* gene amplification or polysomy, that is, pan-*MET* gene amplification. If the copy number of the region including 7q32.1 was increased and the region with increased copy number accounted for >80% of the length of 7q, it was defined as polysomy. The detection process is provided in the Supplementary Data (Supplementary Fig. S2).

### Data Analysis and Statistical Analysis

Progression-free survival (PFS) was analyzed using the Kaplan–Meier method, and patients were censored if their status was not available at the last follow-up.

To differentiate polysomy from normal cases by copy number using NGS, the Youden index was calculated, and the point at the maximum Youden index was considered as the cut-off value. In addition, ROC analysis demonstrated that a cut-off value of 2.3 copies achieved the maximum Youden index in differentiating polysomy from normal copy number.

On the basis of the established cut-off value, the kappa coefficient was calculated for concordance between tissue and plasma assessments of *MET* status using NGS.

Statistical significance was set at *P* < 0.05. All analyses were performed using the IBM SPSS Statistics 24.0 and R 4.2.1.

### Data Availability Statement

Variant Call Format files supporting the findings of this study have been deposited in the Genome Variation Map (GVM) database of National Genomics Data Center under accession number GVM000490 (https://ngdc.cncb.ac.cn/gvm). Other data generated in this study are available within the article and its Supplementary Data. All data generated and analyzed are available from the corresponding author upon reasonable request.

## Results

### Threshold of *MET* Copy Number to Determine Polysomy using NGS

**FIGURE 1:**
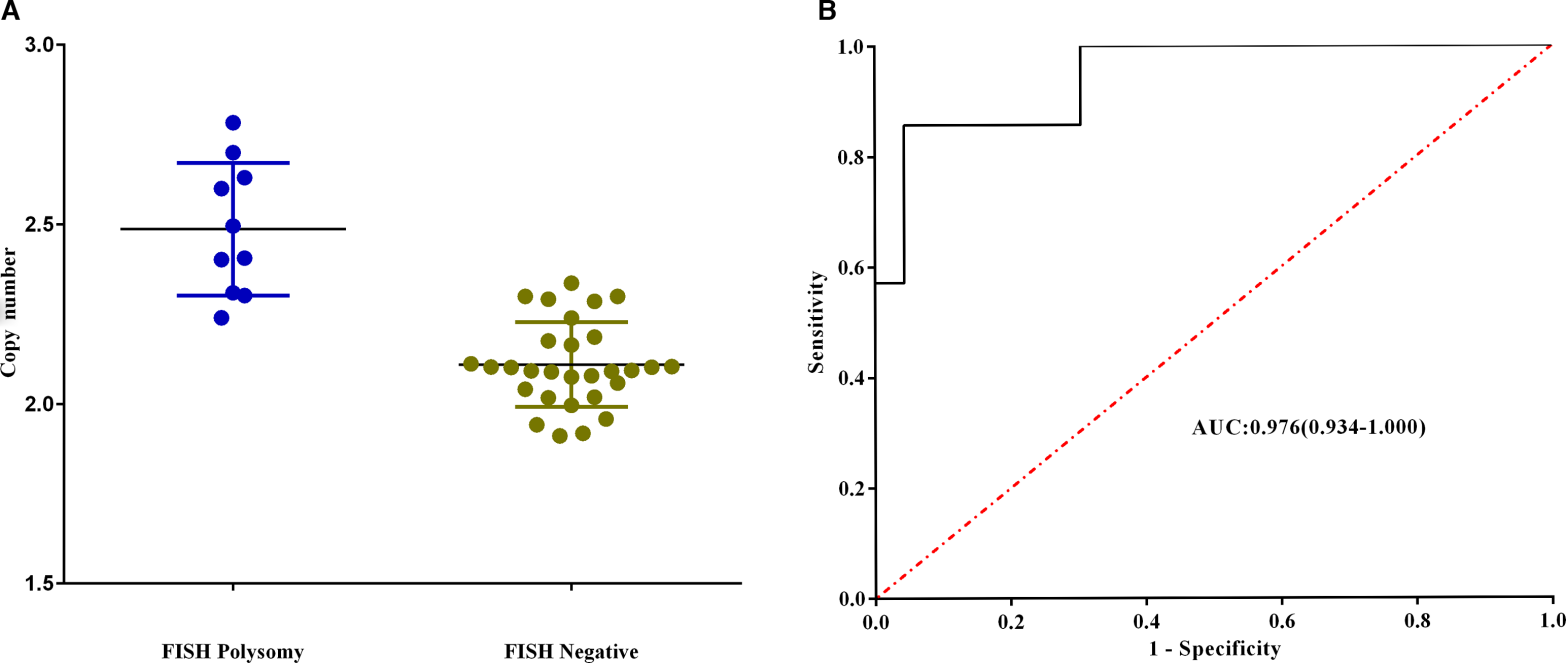
**A,** The distribution of *MET* copy number for FISH polysomy group and FISH negative group. **B,** ROC analysis demonstrated that a cut-off point of 2.3 copies achieved the maximum Youden index for discriminating polysomy from normal copy number (Youden index = 0.866 with a sensitivity of 90% and specificity of 96.6%, AUC = 0.976, and 95% CI = 0.934–1.000, *P* < 0.0001).

Of these 53 patients in cohort 1, 60% (32/53) were male, and the rest (40%, 21/53) were female. The median age of the patients was 57 years, ranging from 31 to 75 years. The predominant histology type was adenocarcinoma (90.6%, 48/53), while 5 patients had adenosquamous carcinoma. Most of the patients were diagnosed with stage IIIA or IIIB (47.2%, 25/53), and the remaining patients had stage II (28.3%, 15/53) and stage IV (24.5%, 13/53). Among the 53 patients with paired tissue FISH and NGS results, 29 cases were classified as normal copy number and 10 cases were classified as polysomy according to FISH status. The distribution of *MET* copy numbers for the two groups is shown in Fig. 1A. ROC analysis also demonstrated that a cut-off point of 2.3 copies achieved the maximum Youden index for discriminating polysomy from normal copy number [Youden index = 0.866 with a sensitivity of 90% and specificity of 96.6%, AUC = 0.976, and 95% confidence interval (CI) = 0.934–1.000, *P* < 0.0001] (Fig. 1B).

### Concordance Between FISH and NGS for Assessment of *MET* Status in Tissue

In total, 53 patients whose tissues were available for both FISH and NGS were enrolled. The FISH and NGS results are presented in Table 1. FISH analysis identified 14 cases of *MET* amplification, 10 cases of polysomy, and 29 cases with normal copy numbers. Using FISH as the gold standard, NGS correctly identified 13 cases with *MET* amplification, nine cases with polysomy, and 28 cases with normal copy numbers. Compared with the FISH test for *MET* amplification, the sensitivity, specificity, and agreement of NGS were 92.9%, 100%, and 98.1%, respectively. Compared with the FISH test for *MET* polysomy, the sensitivity, specificity, and agreement of NGS were 90%, 90%, and 96.2%, respectively. A kappa value of 0.886 also revealed high congruence.

**TABLE 1:** The paired results of FISH and NGS in 53 patients with NSCLC

		*MET* FISH		
		Amplification	Polysomy	Negative
**NGS**	**Amplification**	13	0	0
	**Polysomy**	0	9	1
	**Negative**	1	1	28

Abbreviations: FISH: fluorescence *in situ* hybridization; NGS: next-generation sequencing; *MET*: mesenchymal epithelial transition factor gene.

One *MET*-FISH–positive tumor was identified as negative by NGS. The mean *MET*/CEP7 ratio (10.5/3.9) was 2.7. The copy number was 2.26 copies and no *MET* amplification signal was observed in the CNV map (Supplementary Fig. S3). One *MET*-FISH polysomy tumor was classified as negative using the NGS assay. The *MET*/CEP7 ratio (5.1/2.7) was 1.9, while the copy number derived from NGS was 2.24 (Supplementary Fig. S4). One *MET*-FISH–negative patient was classified as having *MET* polysomy by NGS. The *MET*/CEP7 ratio (3.6/2.2) was 1.6, and copy number was 2.5596 (Supplementary Fig. S5). The tumor cell content was 15%, 11%, and 37%, respectively. Additional details are provided in the Supplementary Materials and Methods.

### Gene Mutation Profiles in Patients with NSCLC with Different *MET* Status

**FIGURE 2:**
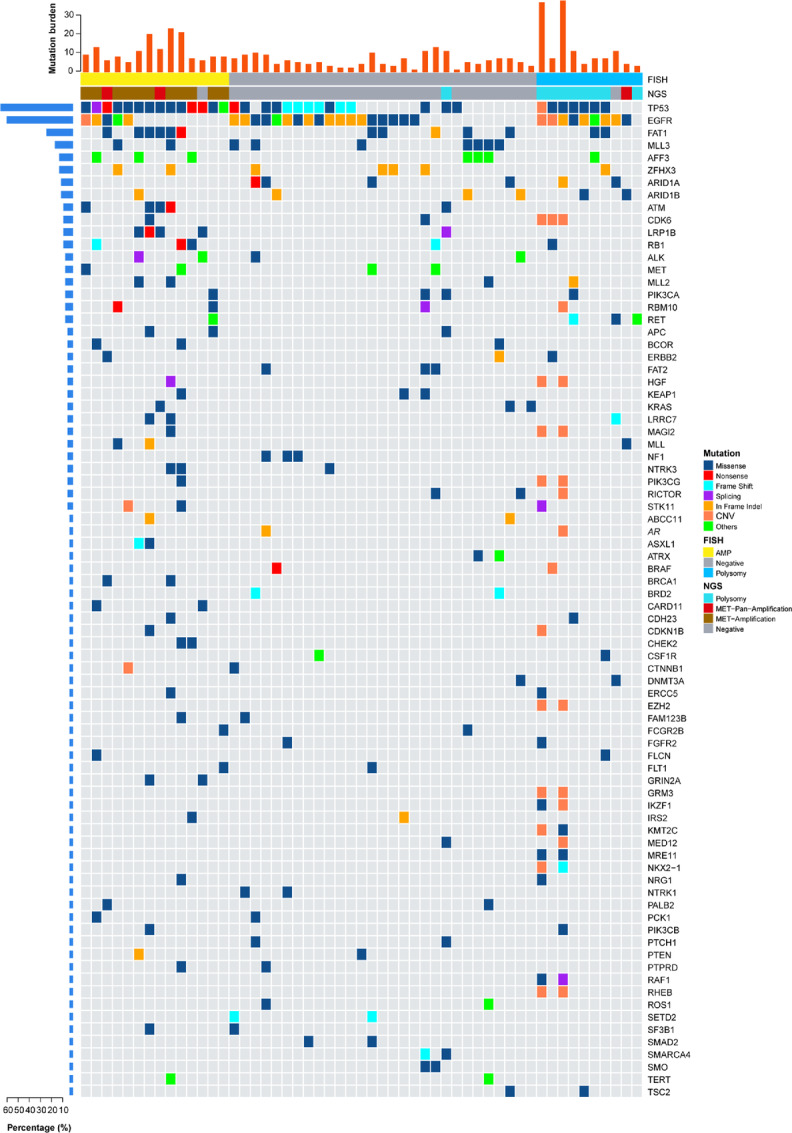
*MET* status of 53 patients determined by FISH and NGS, overlapping oncogenes, and mutation burden.

Figure 2 shows the *MET* status of 53 patients determined by FISH and NGS, overlapping oncogenes, and mutation burden. Fewer comutations of oncogenes were detected in the *MET* amplification group than in the other two groups. The frequencies of overlapping oncogenes were similar in the polysomy and normal copy number groups.

### Concordance Between Tissue and Plasma-based Testing for Assessment of *MET* Status Using NGS

The plasma and tissue NGS results were compared for 261 patients who underwent concurrent testing, and the details are listed in Table 2. On the basis of the established standard, NGS identified 19 cases of *MET* amplification, eight cases of pan-*MET* amplification, 48 cases of polysomy, and 186 cases of normal copy number in tissue. The results of NGS in the plasma were consistent with those of gene detection in tissue from 10 patients with *MET* amplification, 2 patients with pan-*MET* amplification, 6 patients with polysomy, and 181 patients with normal copy number (Table 2). The sensitivity and specificity of NGS for detecting *MET* amplification using NGS were 53% and 100%, respectively. The concordance between the amplification detection rates in circulating tumor DNA (ctDNA) and tissue samples was 96.6%. For polysomy detection using NGS, sensitivity and specificity were 12.5% and 50%, respectively. The concordance rate between the two sample types was 90.7%.

**TABLE 2:**
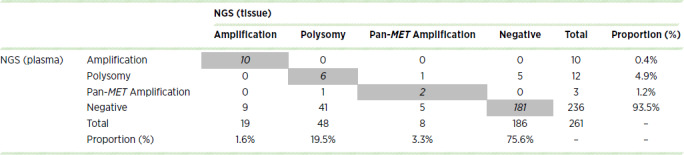
The plasma and tissue NGS results of 261 patients who underwent concurrent testing

**FIGURE 3:**
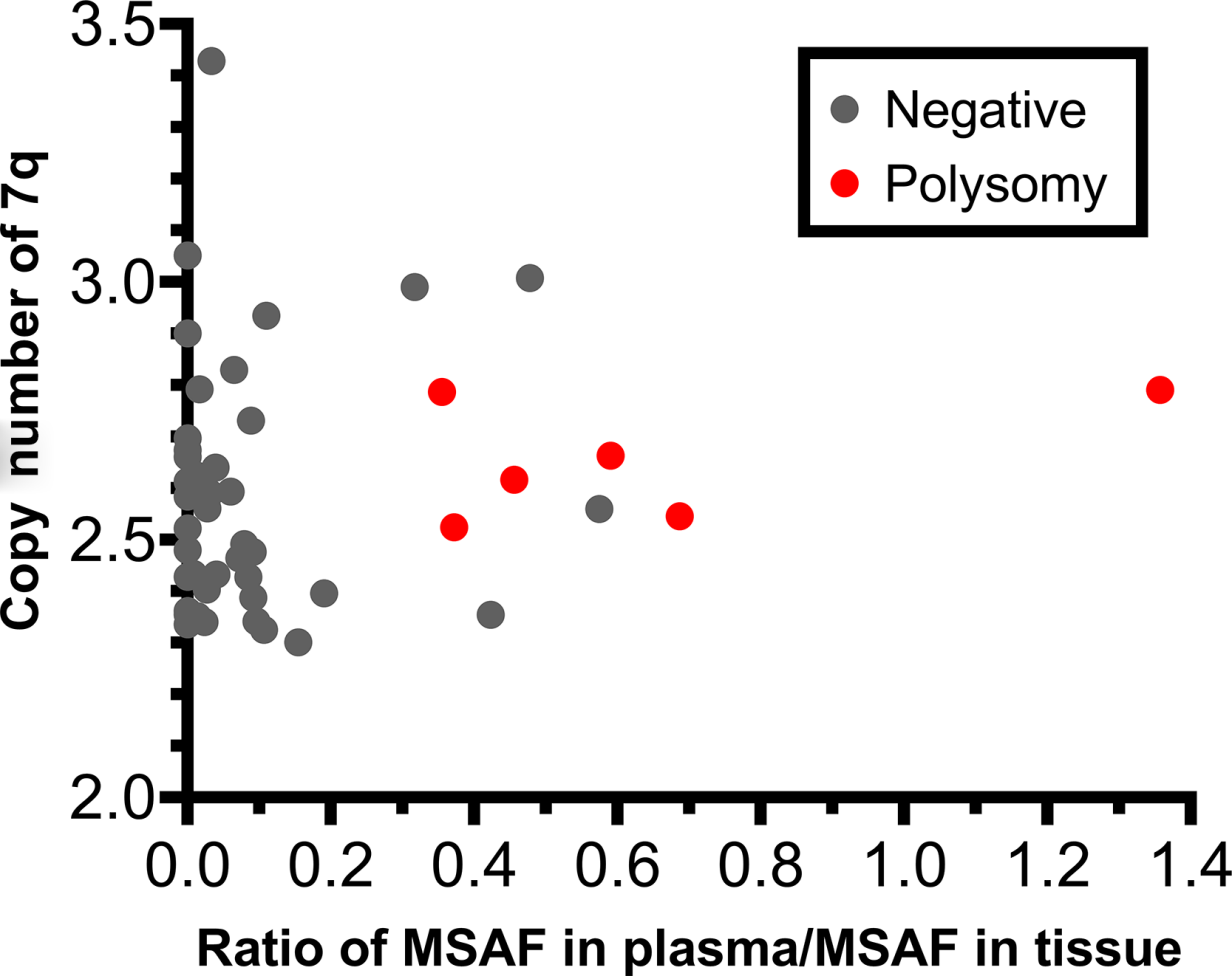
Distribution of the 7q copy numbers in tissue and ratio of MSAF in plasma to those in the tissues of each polysomy case determined by NGS.

Figure 3 shows the 7q copy numbers in tissue and ratio of maximum somatic allele frequency (MSAF) in plasma to those in the tissues of each polysomy case determined by NGS. In 48 patients, polysomy was consistently detected in the plasma of 6 patients. All 6 patients presented a relatively high copy number of 7q (>2.5) in tissues, and their ratio of MSAF in plasma to that in tissue was relatively high (>0.35). Among the other 42 patients who were negative, 38 presented with a low plasma mutation frequencies/tissue mutation frequency ratio (<0.25).

On the basis of these results, we calculated the detection rates of liquid biopsies in selected patients. First, the *EGFR* mutation rate was higher than 5% in 45 patients. Among the 45 patients, *MET* amplification was identified by NGS in the tissues of 6 patients. Among the 6 patients, 3 presented with *MET* amplification in plasma ctDNA. When limited to 10 patients with a MSAF higher than 5%, *MET* amplification was detected in plasma ctDNA from 7 patients. Comparison of ctDNA with tissue in detecting *MET* amplification using NGS identified a 70% positive percent agreement (PPA) and 95% negative percent agreement (NPA) after optimization.

Of the 48 patients in whom polysomy was identified by NGS in tissue, *EGFR* in ctDNA was found to be mutated at a frequency higher than 0.5% in 8 patients. Among the 8 patients, only 1 presented with polysomy in plasma ctDNA. Among the 12 patients with MSAF > 5%, polysomy was detected in plasma ctDNA from 5 patients. Following our optimization, the sensitivity and specificity of NGS for defining polysomy with plasma according to different ctDNA mutation frequencies were 42% and 63%, respectively. The concordance rate between the two sample types for detecting polysomy was 85% after optimization.

### *MET* Status Identified Using NGS in *MET*-high Patients and its Clinical Relevance for *MET* Inhibitors

Between June 2018 and November 2021, 46 patients with NSCLC with a high *MET* copy number assessed by NGS were enrolled in the study. Detailed characteristics of the recruited patients are listed in Supplementary Materials and Methods (Supplementary Table S1). All patients received *MET* inhibitors, such as crizotinib or savolitinib, as second- or third-line therapies. The partial response (PR) rate was 35% and the median PFS was 2.6 months. The PR rate was significantly higher in the *MET* amplification group than in the polysomy group (50% vs. 21.4%, *P* < 0.001), whereas there was no significant difference in the PR rate between the polysomy and normal groups (21.4% vs. 25%, *P* = 0.77). The median PFS was 5.9 months in the *MET* amplification group, which was significantly higher than that in the polysomy group (2 months, *P* < 0.001). The median PFS was similar between the polysomy and normal groups (2 vs. 1.9 months, *P* = 0.79; Fig. 5). Summary of MET status tested by NGS, MET inhibitors with clinical outcome and dynamic changes in gene alterations was listed in Supplementary Table S2.

## Discussion

*MET* amplification is regarded as an oncogenic driver and represents a therapeutic target (2, 3, 14). However, *MET* amplification comprises a minority of patients with positive FISH results or high *MET*. Polysomy was detected on chromosome 7 in other patients with *MET* CNG. However, to date, no unified criteria have been established to define polysomy using copy numbers derived from NGS. In several studies, the FISH and NGS detection results of *MET* status were in poor concordance with each other (13, 15). There is no established cut-off value to define polysomy, and whether it is correlated with the response to *MET* inhibitors remains to be investigated. In this study, we established a new methodology for detecting *MET* polysomy using NGS. Moreover, our study showed the poor efficacy of *MET* inhibitors in the treatment of patients with *MET* polysomy.

NGS technology has improved gene mutation detection and enables more appropriate use of targeted drug therapies (11, 16). However, for *MET* detection, some issues remain unresolved with NGS. For instance, due to methodologic problems in previous NGS assays, it is challenging to discriminate between *MET* amplification and polysomy in *MET*-high patients. Polysomy is occasionally misclassified as *MET* amplification, and this would include more patients; therefore, some patients might show little clinical benefit to *MET*-targeted therapy, leading to a diluted clinical benefit. In our study, we proposed a specific algorithm after sequencing to detect the *MET* status, which is capable of discriminating polysomy from *MET* amplification. As the first exploratory study on the topic, the optimum cut-off value on which future research can be based should be determined. The threshold copy number for polysomy was determined using the ROC curve. Using the Youden index, we found that the appropriate cutoff was 2.3 with a sensitivity of 90% and specificity of 96.6%.

**FIGURE 4:**
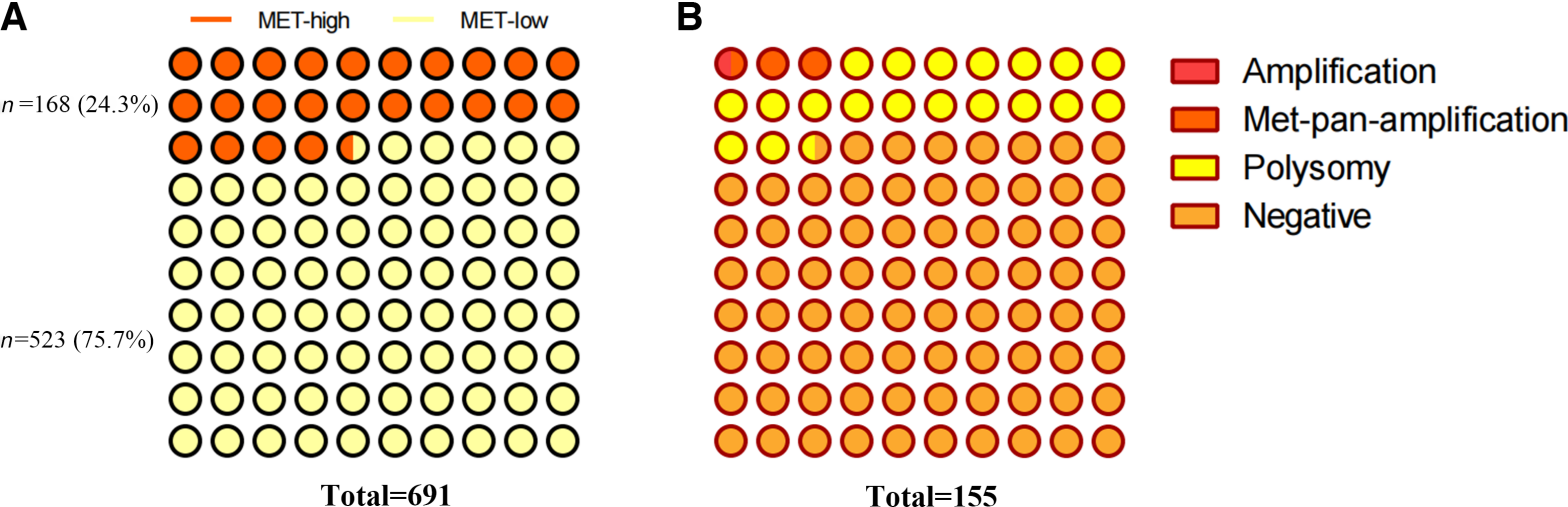
Prevalence of *MET* high in treatment-naïve metastatic NSCLC. Prevalence of *MET* statuses in treatment-naïve metastatic *EGFR*-mutant NSCLC.

On the basis of this cut-off value, *MET*-high and *MET*-low accounted for 24.3% and 75.7% in our in-house cohort of 691 patients with untreated NSCLC (Fig. 4A.) *MET* amplification, pan-*MET* amplification, and polysomy accounted for 1%, 3%, and 19%, respectively, in our in-house cohort of 155 patients with untreated *EGFR*-mutated NSCLC (Fig. 4B). This proportion was similar to that of patients with untreated *EGFR*-mutated NSCLC reported in previous studies (12), which verified the rationality of the threshold to some extent.

**FIGURE 5:**
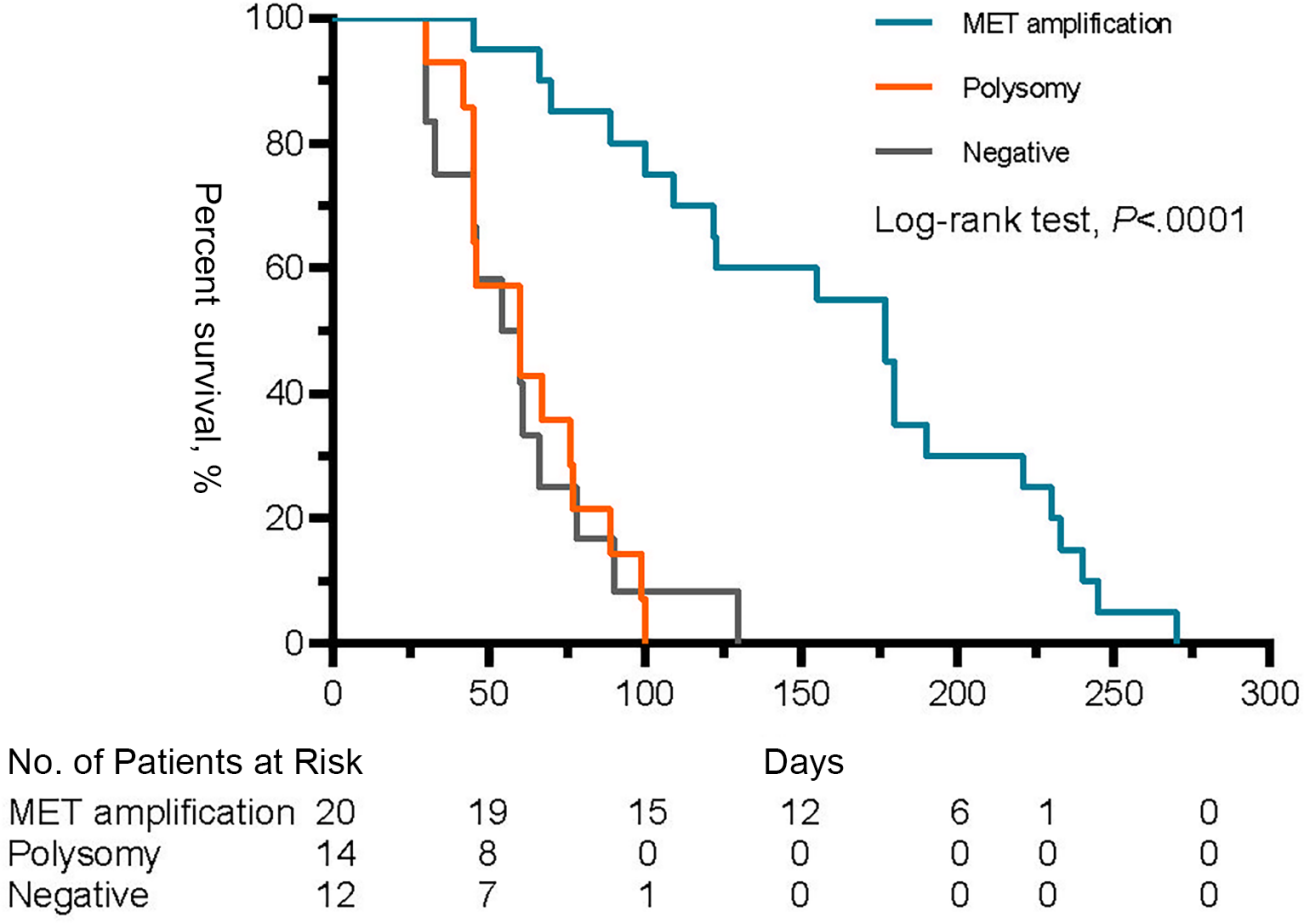
PFS between *MET* amplification, polysomy, and negative by NGS (*n* = 46).

FISH testing for tissue is commonly used in the detection of *MET* abnormalities (17). Both *MET* amplification and polysomy can be detected by FISH, and it has been considered the gold standard for *MET* amplification detection. In the current study, the algorithm and copy-number cut-off value for polysomy were applied to distinguish the different *MET* statuses in tissue using NGS. Fortunately, we found satisfactory concordance between FISH and NGS assays for testing both *MET* amplification and polysomy, especially in the former. Our results showed higher PPA (86% vs. 4%), positive predictive value (PPV; 86% vs. 50%), and negative predictive value (NPV; 96% vs. 68%) in detecting polysomy than those reported in the TATTON study, demonstrating that the proposed approach has better detection performance (18). In general, there was substantial concordance between the two methods for all three groups, with a kappa value of 0.886. Up to now, FISH is considered to be the gold standard for detecting MET amplification. There is no uniform interpretation standard for MET gene amplification detected by FISH. NGS is constantly developing and becoming more accessible. The limitation is that the data quality is highly dependent on the platform used, and the lower copy number (especially when the tumor cell content is low) may not be detected. In our study, the FISH and NGS detection results of *MET* status were in good concordance with each other, which lay the foundation for NGS as routine method to detect *MET* aberrant molecular alterations. What's more, NGS based on liquid biopsy may be an alternative when tissue biopsy is not available.

Liquid biopsy can overcome many limitations of conventional solid biopsy and has become a valid alternative to tissue biopsy (19). Liquid biopsy is noninvasive and safe and can be repeated easily. Moreover, it is expected to reveal the entire molecular profile of a patient's malignancy. It has been widely believed that liquid biopsy has the potential to change the paradigm in the management of patients with cancer (20). There is growing evidence to support the clinical use of plasma ctDNA to detect multiple gene aberrations by NGS. Recently, the International Association for the Study of Lung Cancer updated consensus for liquid biopsy use in NSCLC. It recommends that plasma ctDNA be considered a valid method for genotyping of newly diagnosed patients with advanced NSCLC. At the time of acquired resistance after TKI therapy, initial use of ctDNA is preferred to evaluate the mechanisms of resistance (21). Our study also explored the diagnostic performance of liquid biopsy in detecting *MET* abnormalities in patients with NSCLC. The results showed that liquid biopsy had a high NPV and high specificity for detecting *MET* amplification. NPV and specificity were lower for polysomy detection. In agreement with previous studies, liquid biopsy has a moderate PPV for detecting polysomy (15). In patients with MSAF higher than 5%, the detection rates of both *MET* amplification and polysomy were higher than those in unselected patients.

Following a case-by-case analysis, we found that a higher copy number of *MET* in tissues and higher ctDNA content can increase the detection rate of polysomy in plasma. In addition, we suppose that the degree of intratumoral heterogeneity can also influence the detection rate of polysomy. Therefore, the generally good concordance between tissue and plasma for testing *MET* amplification based on NGS in this study highlights the feasibility of liquid biopsy as an alternative to tissue testing for *MET* detection.

It has been previously suggested that the response to crizotinib, an inhibitor of c-*MET* activity, may determine which type of testing is the most relevant in predicting the response to *MET* inhibition (22). For reliable means of detection, the response to crizotinib could distinguish which type of *MET* status acts as a tumor driver gene. According to our study, we found significant survival benefits among patients with *MET* amplification detected using NGS with a *MET* inhibitor; however, there was no significant benefit in both polysomy and normal copy groups with similar PR rates and PFS. This finding demonstrates that polysomy may not be a therapeutic target for *MET* inhibitors. A few oncogene overlaps were observed in the *MET* amplification group, whereas gene mutation profiles were comparable between the polysomy and negative groups. This finding is consistent with polysomy, which does not act as an oncogenic driver. Multivariate analysis revealed that PFS was associated only with *MET* status, other than *MET* copy number, which is consistent with a previous study (12).

In summary, the reasonable cut-off value to distinguish polysomy and normal copy number may be 2.3 copies. Identification of amplification and polysomy depends on the different proportion of the amplified region on the chromosome 7q. Table 3 shows the definition/cut-off value of the different *MET* aberrations. Our results indicate that NGS may serve as an alternative method for detecting *MET* amplification or polysomy in NSCLC tissues. Liquid biopsy could serve as a substitute for tissue biopsy for detecting *MET* amplification using NGS. It appears that polysomy does not act as a true oncogenic driver, even though it has a high *MET* copy number. These findings guaranteed further testing and validation in cohort with enlarged sample size.

**TABLE 3:** Cut-off value of the different *MET* aberrations

*MET* GCN			
<2.3	≥2.3		
Negative	Proportion of the amplified region on the chromosome 7q		
	≤10%	10%∼80%	≥80%
	*MET*-amplification	pan-*MET* amplification	Polysomy

Abbreviations: GCN: gene copy number; *MET:* mesenchymal epithelial transition factor gene.

As the first exploratory study to establish a methodology for detecting polysomy using NGS, our results lay the foundation for future studies on the same topic. Furthermore, our study also demonstrated the feasibility of detecting *MET* status using NGS. However, this study has some limitations. First, it had a relatively small sample size. Second, its retrospective nature and the fact that the results were from one institution without an external validation group are obvious limitations. Third, most *MET* inhibitors used in our cohort were crizotinib, and whether the poor response in patients with *MET* polysomy might be related to drug accessibility needs to be explored.

## Supplementary Material

Table TS1 Clinicopathologic characteristics of patients in cohort 3.

Figure S1 Flowchart of the study design

Table TS2 Summary of MET status tested by NGS, MET inhibitors with clinical outcome and dynamic changes in gene alterations in cohort 3

Figure S2 The detection process using NGS of the study.

Figure S3 One MET-FISH-positive tumor was identified as negative by NGS

Figure S4 One MET-FISH polysomy tumor was classified as negative using the NGS assay.

Figure S5 One MET-FISH-negative patient was classified as having MET polysomy by NGS
