# Supplementary material for: Detection of MET Polysomy by Next-generation Sequencing and Its Clinical Relevance for MET Inhibitors
Source: Cancer Res Commun. 2023 Apr 4;3(4):532–9. doi: 10.1158/2767-9764.CRC-22-0438 (PMC10072163; doi:10.1158/2767-9764.CRC-22-0438)
Supplement: Table TS1 — Clinicopathologic characteristics of patients in cohort 3. [file crc-22-0438-s01.doc]

**Table S1.** Clinicopathologic characteristics of patients in cohort 3.

| Characteristics | N (%) |
| --- | --- |
| Age |  |
| <60 years old | 21 |
| ≥60 years old | 25 |
| Gender |  |
| Female | 26 |
| Male | 20 |
| MET status by FISH |  |
| Amplification | 24 |
| Polysomy | 16 |
| Negative | 6 |
| MET status by NGS |  |
| Amplification | 20 |
| Polysomy | 14 |
| Negative | 12 |
| MET GCN |  |
| GCN≥5 | 17 |
| GCN<5 | 29 |
