# Supplementary figures and images for: Detection of MET Polysomy by Next-generation Sequencing and Its Clinical Relevance for MET Inhibitors
Source: Cancer Res Commun. 2023 Apr 4;3(4):532–9. doi: 10.1158/2767-9764.CRC-22-0438 (PMC10072163; doi:10.1158/2767-9764.CRC-22-0438)

Figure S1

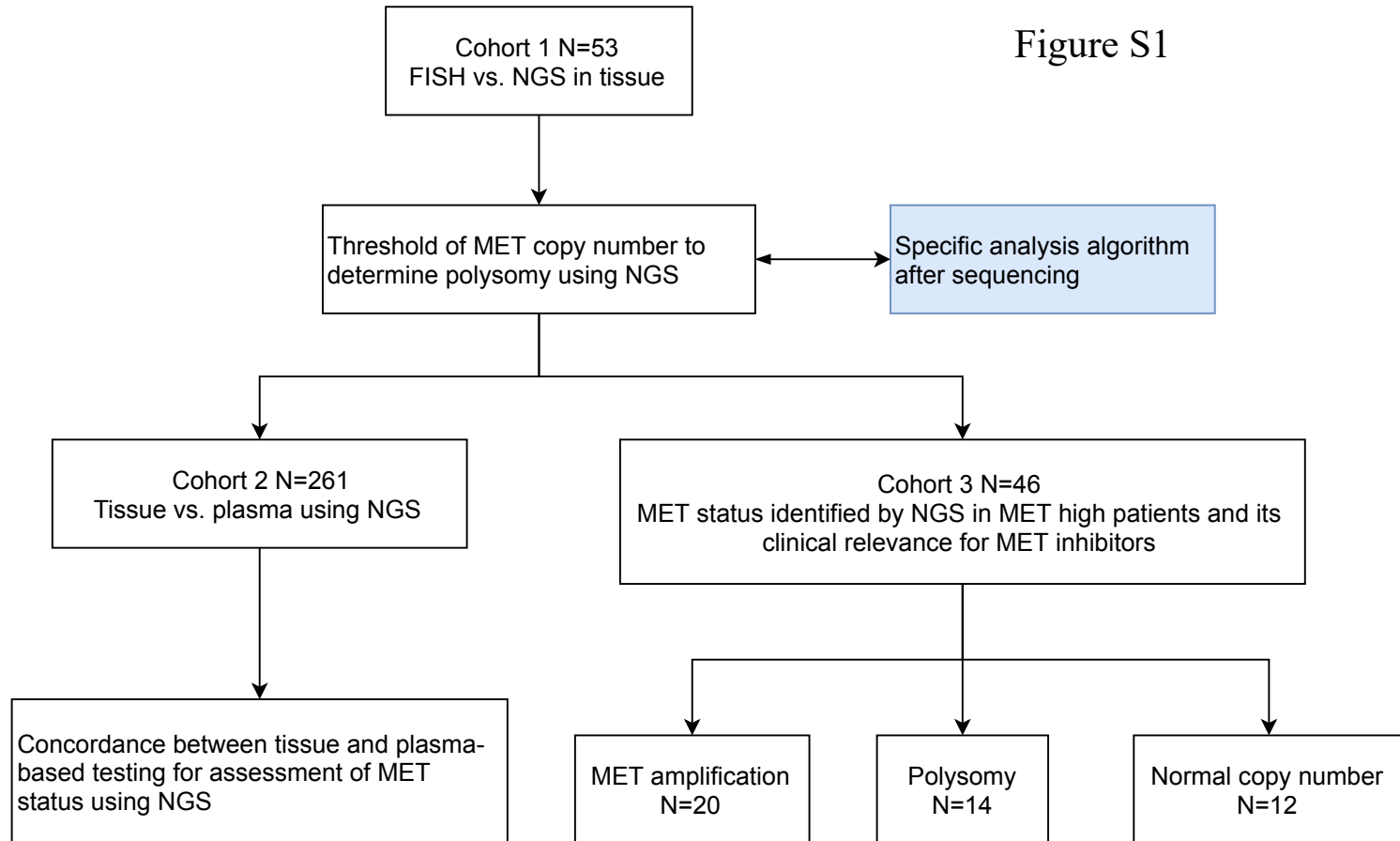

Flowchart of the study design

Supplement: Figure S1 — Flowchart of the study design [file crc-22-0438-s02.pdf]

Figure S2

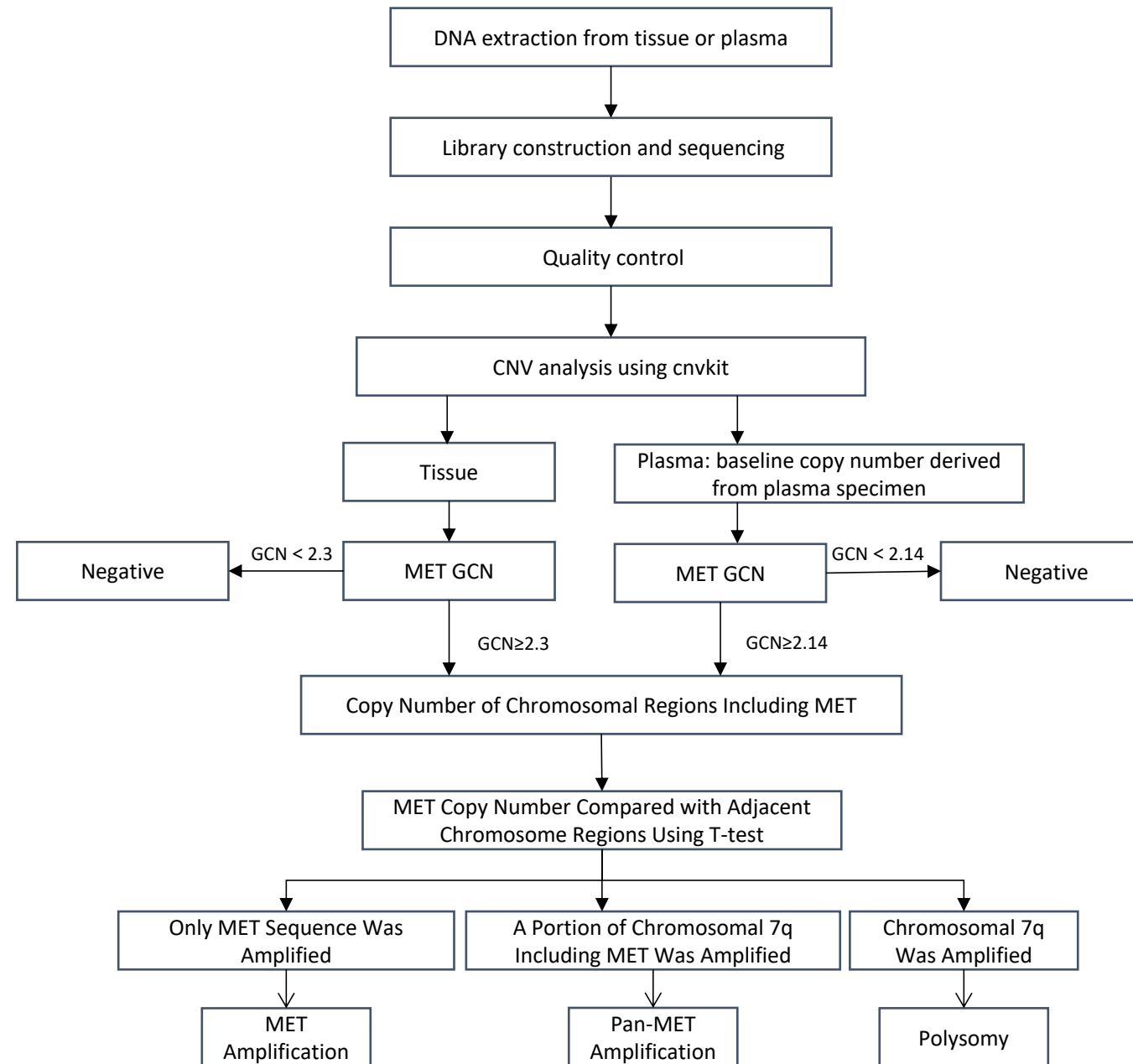

The detection process using NGS of the study

Supplement: Figure S2 — The detection process using NGS of the study. [file crc-22-0438-s04.pdf]

Figure S3

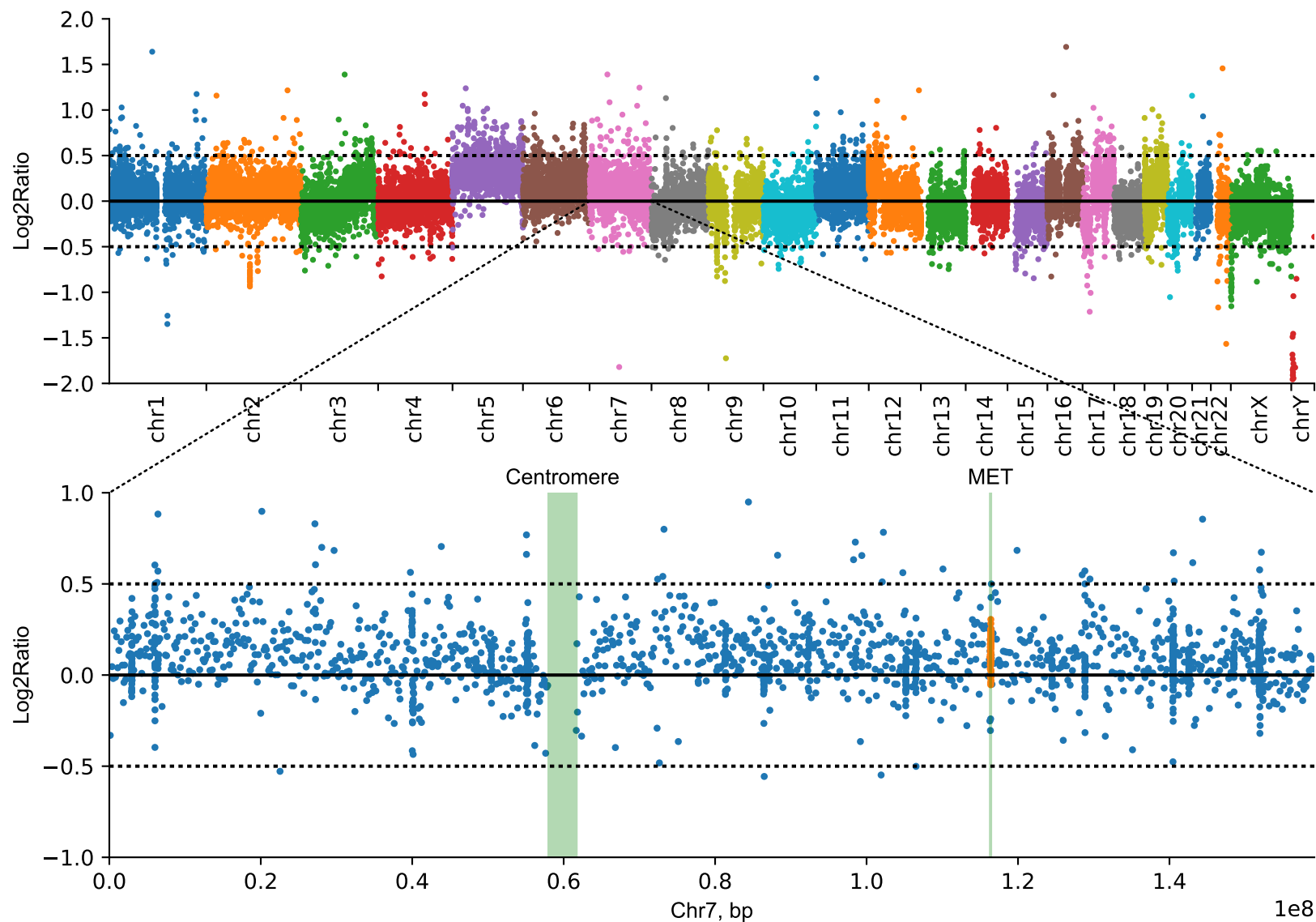

One MET-FISH-positive tumor was identified as negative by NGS

Supplement: Figure S3 — One MET-FISH-positive tumor was identified as negative by NGS [file crc-22-0438-s05.pdf]

Figure S4

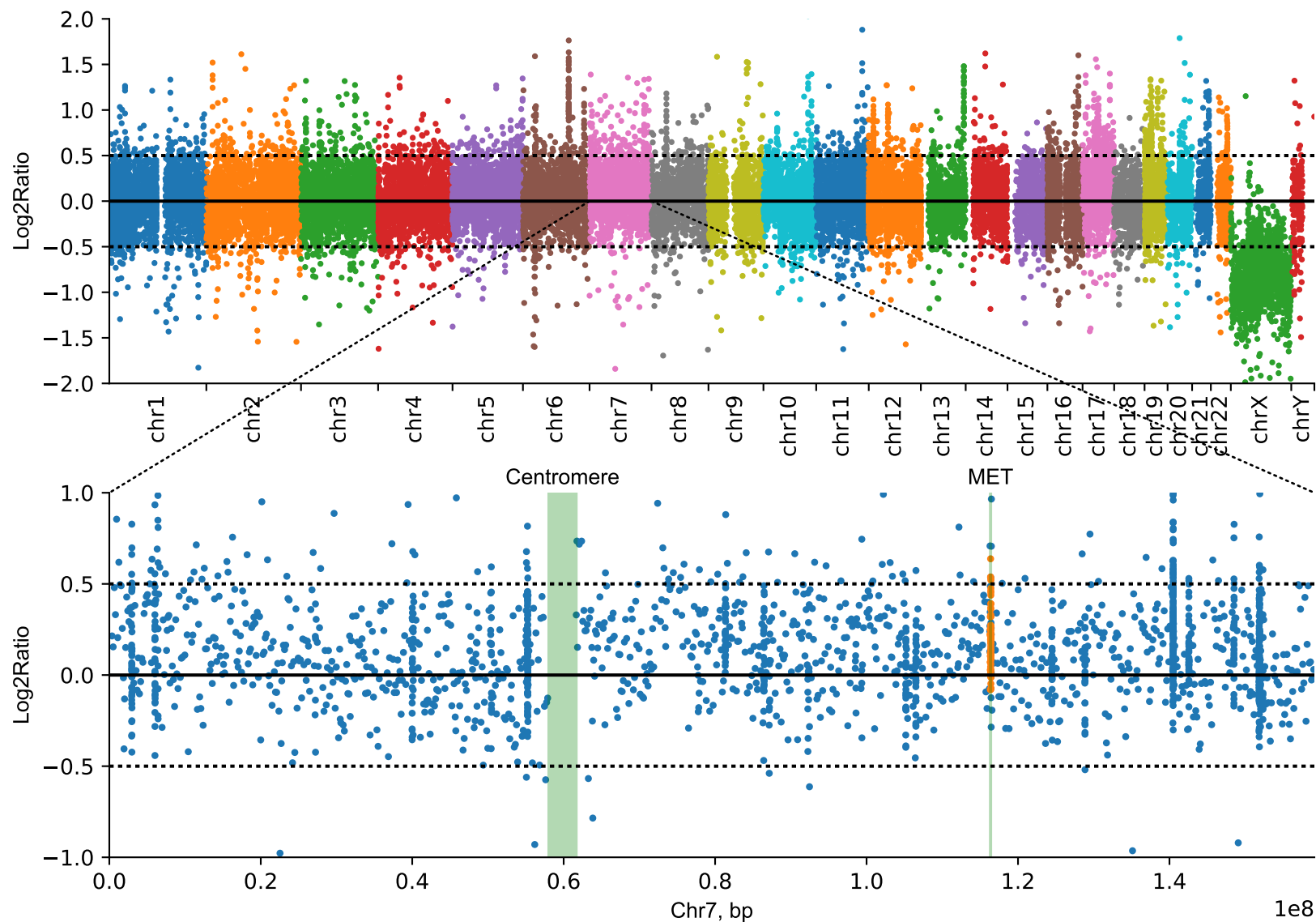

One MET-FISH polysomy tumor was classified as negative using the NGS assay

Supplement: Figure S4 — One MET-FISH polysomy tumor was classified as negative using the NGS assay. [file crc-22-0438-s06.pdf]

Figure S5

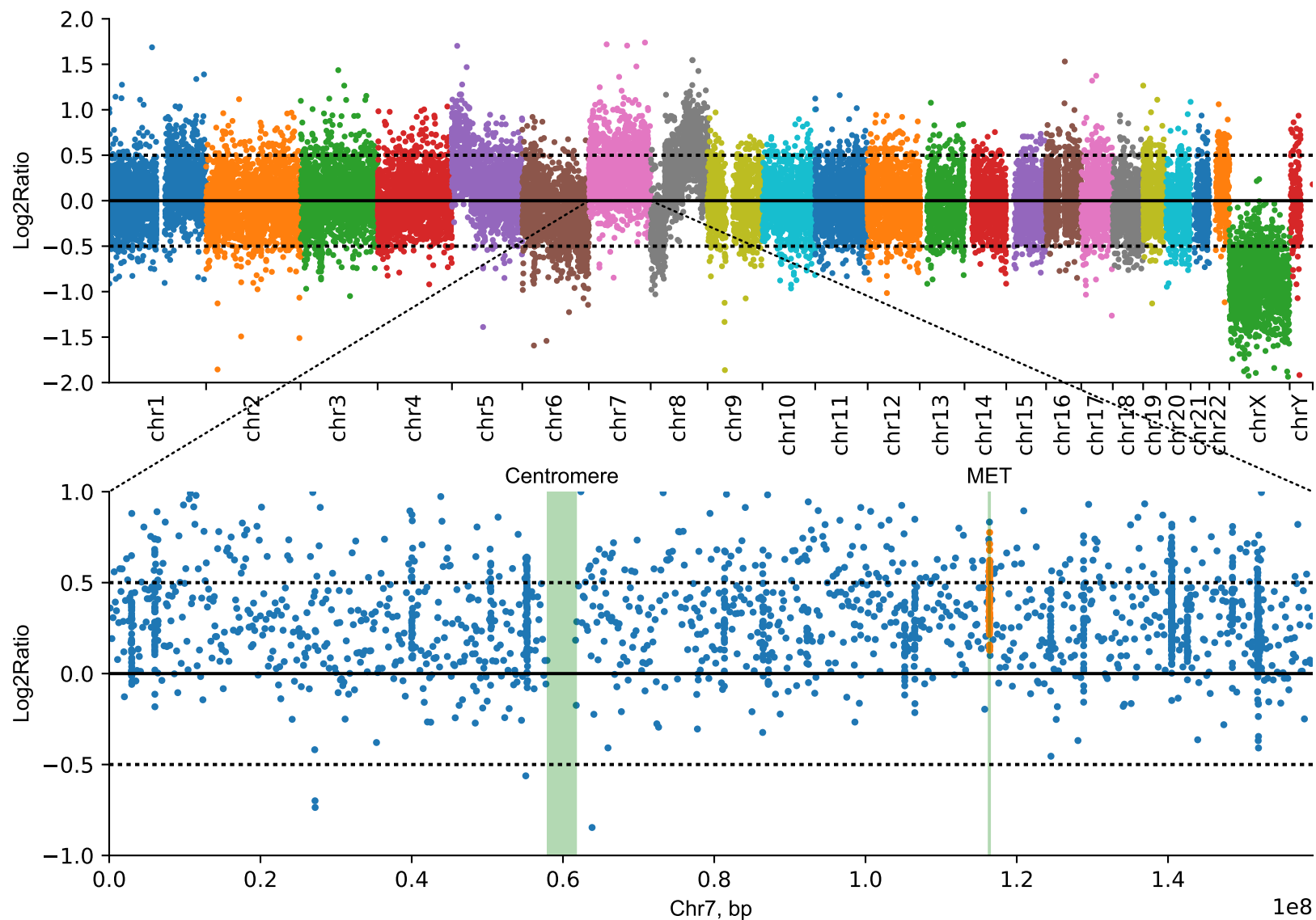

One MET-FISH-negative patient was classified as having MET polysomy by NGS

Supplement: Figure S5 — One MET-FISH-negative patient was classified as having MET polysomy by NGS [file crc-22-0438-s07.pdf]
