# Supplementary material for: Detection of MET Polysomy by Next-generation Sequencing and Its Clinical Relevance for MET Inhibitors
Source: Cancer Res Commun. 2023 Apr 4;3(4):532–9. doi: 10.1158/2767-9764.CRC-22-0438 (PMC10072163; doi:10.1158/2767-9764.CRC-22-0438)
Supplement: Table TS2 — Summary of MET status tested by NGS, MET inhibitors with clinical outcome and dynamic changes in gene alterations in cohort 3 [file crc-22-0438-s03.doc]

Table S2. Summary of *MET* status tested by NGS, *MET* inhibitors with clinical outcome and dynamic changes in gene alterations

| Patient | *MET* status detected by NGS | *MET* GCN | Pretreatment mutation | *MET*-targeted therapy regimen | Number of prior treatment lines | PFS  (mo) | Post-treatment mutation |
| --- | --- | --- | --- | --- | --- | --- | --- |
| 1 | Amplification | 15.1 | *EGFR* 19del | crizotinib | 1 | 9 | *MET*-amplification;  *EGFR* 19del |
| 2 | Amplification | 6.3 | *EGFR* L858R | savolitinib | 1 | 7.7 | ND |
| 3 | Amplification | 7.4 | *EGFR* L858R | crizotinib | 1 | 6 | *MET*-amplification |
| 4 | Amplification | 11.2 | *EGFR* 19del;  *EGFR* T790M | crizotinib | 2 | 3.3 | *EGFR* T790M |
| 5 | Amplification | 8.7 | *EGFR* 19del | crizotinib | 1 | 1.5 | ND |
| 6 | Amplification | 14.7 | ND | crizotinib | 2 | 8 | ND |
| 7 | Amplification | 6.9 | ND | crizotinib | 1 | 5.9 | ND |
| 8 | Amplification | 6.8 | ND | crizotinib | 1 | 6.3 | ND |
| 9 | Amplification | 9.2 | *TP53* R273L | crizotinib | 1 | 2.2 | ND |
| 10 | Amplification | 9.8 | *TP53* R248W;  *CDK6* amplification | crizotinib | 1 | 2.3 | ND |
| 11 | Amplification | 4.4 | *EGFR* L858R;  *Rb1* deletion | savolitinib | 1 | 3.6 | ND |
| 12 | Amplification | 8.6 | ND | crizotinib | 1 | 7.4 | ND |
| 13 | Amplification | 12.6 | ND | crizotinib | 2 | 4 | ND |
| 14 | Amplification | 14.5 | ND | crizotinib | 1 | 5.2 | ND |
| 15 | Amplification | 13.6 | ND | crizotinib | 1 | 6 | ND |
| 16 | Amplification | 4.8 | ND | crizotinib | 1 | 8.2 | ND |
| 17 | Amplification | 13.8 | *TP53* S127F;  *SMARCA4* 20exon | crizotinib | 1 | 7.8 | *TP53* S127F |
| 18 | Amplification | 11.6 | *NTRK3* | crizotinib | 1 | 4.1 | ND |
| 19 | Amplification | 10.6 | *TP53* Q317*;  *CDKN2A* splicing | savolitinib | 1 | 5.9 | *TP53* Q317* |
| 20 | Amplification | 4.76 | *MET* exon14 skipping | crizotinib | 1 | 3 | *MET*-amplification;  *TP53* exon4 |
| 21 | Polysomy | 4.7 | *KRAS* G12C | crizotinib | 1 | 2 | *KRAS* G12C |
| 22 | Polysomy | 4.55 | *TP53* R280I;  *MAP3K1* | crizotinib | 2 | 3 | ND |
| 23 | Polysomy | 4.18 | *MET* 19exon | crizotinib | 1 | 3.3 | ND |
| 24 | Polysomy | 4.8 | *TP53* exon4 | capmatinib | 1 | 3.3 | ND |
| 25 | Polysomy | 3.96 | *EGFR* 20ins | crizotinib | 1 | 1.5 | *EGFR* 20ins |
| 26 | Polysomy | 3.6 | *EGFR* L858R | crizotinib | 1 | 1.6 | ND |
| 27 | Polysomy | 3.4 | *EGFR* 19del | crizotinib | 2 | 2.2 | ND |
| 28 | Polysomy | 4.8 | *EGFR* L858R;  *TP53* R273L | crizotinib | 1 | 2.5 | ND |
| 29 | Polysomy | 4.1 | *EGFR* L861Q | crizotinib | 1 | 2.6 | ND |
| 30 | Polysomy | 3.6 | *KRAS* G12C | crizotinib | 1 | 2 | *KRAS* G12C |
| 31 | Polysomy | 3.64 | *EGFR* L858R | crizotinib | 1 | 1.5 | ND |
| 32 | Polysomy | 2.82 | *EGFR* 19del | crizotinib | 1 | 1 | ND |
| 33 | Polysomy | 2.46 | *EGFR* 20ins | crizotinib | 1 | 1.4 | ND |
| 34 | Polysomy | 2.8 | ND | crizotinib | 1 | 1.5 | ND |
| 35 | Negative | 2.78 | *EGFR* 19del | crizotinib | 1 | 1.5 | *EGFR* 19del |
| 36 | Negative | 2.6 | *EGFR* L858R | crizotinib | 1 | 1.8 | ND |
| 37 | Negative | 2.36 | *EGFR* G719C | crizotinib | 1 | 1.1 | ND |
| 38 | Negative | 2.66 | *KRAS* G12C | crizotinib | 1 | 1 | ND |
| 39 | Negative | 2.82 | *EGFR* S768I;  TP53 | crizotinib | 1 | 1 | ND |
| 40 | Negative | 3.4 | *EGFR* L858R | crizotinib | 1 | 1.5 | ND |
| 41 | Negative | 2.23 | *EGFR* 19del | crizotinib | 1 | 2 | ND |
| 42 | Negative | 2.17 | *EGFR* L858R;  *EGFR* T790M | crizotinib | 1 | 4.3 | *EGFR* T790M |
| 43 | Negative | 2.28 | *EML4-ALK* | crizotinib | 1 | 3 | ND |
| 44 | Negative | 2.31 | *ROS1-CD74* | crizotinib | 1 | 2.2 | ND |
| 45 | Negative | 2.11 | *EGFR* 19del | crizotinib | 2 | 2.6 | ND |
| 46 | Negative | 1.91 | *KRAS* G12V | crizotinib | 1 | 2 | ND |

Abbreviation: FISH: fluorescence in situ hybridization; NGS: next generation sequencing; MET: mesenchymal epithelial transition factor gene; GCN: gene copy number; ND: not detected; PFS: progression-free survival
